# Loss of the transcriptional repressor Rev-erbα upregulates metabolism and proliferation in cultured mouse embryonic fibroblasts

**DOI:** 10.1038/s41598-021-91516-5

**Published:** 2021-06-11

**Authors:** Sean P. Gillis, Hongwei Yao, Salu Rizal, Hajime Maeda, Julia Chang, Phyllis A. Dennery

**Affiliations:** 1 Department of Molecular Biology, Cellular Biology, and Biochemistry, Brown University, 185 Meeting Street, Providence, RI 02912 USA; 2 Department of Pediatrics, Warren Alpert Medical School of Brown University, 593 Eddy St Suite 125, Providence, RI 02903 USA

**Keywords:** Molecular biology, Molecular medicine

## Abstract

The transcriptional repressor Rev-erbα is known to down-regulate fatty acid metabolism and gluconeogenesis gene expression. In animal models, disruption of Rev-erbα results in global changes in exercise performance, oxidative capacity, and blood glucose levels. However, the complete extent to which Rev-erbα-mediated transcriptional repression of metabolism impacts cell function remains unknown. We hypothesized that loss of Rev-erbα in a mouse embryonic fibroblast (MEF) model would result in global changes in metabolism. MEFs lacking Rev-erbα exhibited a hypermetabolic phenotype, demonstrating increased levels of glycolysis and oxidative phosphorylation. Rev-erbα deletion increased expression of hexokinase II, transketolase, and ribose-5-phosphate isomerase genes involved in glycolysis and the pentose phosphate pathway (PPP), and these effects were not mediated by the transcriptional activator BMAL1. Upregulation of oxidative phosphorylation was not accompanied by an increase in mitochondrial biogenesis or numbers. Rev-erbα repressed proliferation via glycolysis, but not the PPP. When treated with H_2_O_2_, cell viability was reduced in Rev-erbα knockout MEFs, accompanied by increased ratio of oxidized/reduced NADPH, suggesting that perturbation of the PPP reduces capacity to mount an antioxidant defense. These findings uncover novel mechanisms by which glycolysis and the PPP are modulated through Rev-erbα, and provide new insights into how Rev-erbα impacts proliferation.

## Introduction

Rev-erbα is a nuclear receptor and transcriptional repressor that forms a key component of the core circadian clock. Rev-erbα represses expression of *Arntl*, a gene encoding the transcriptional activator BMAL1 through competition for ROR response element (RORE) binding with the transcriptional activator RORα. Thus, Rev-erbα can influence target gene expression through direct binding of ROREs and RevDR2 motifs or through its indirect effect on BMAL1/CLOCK transcriptional activation via E-Box elements^1–5^. This transcription-translation factor feedback loop regulates expression of a significant portion of the genome. Amongst the processes that are controlled by this mechanism are metabolism, the oxidative stress response, and proliferation^4,6^.

In hepatocytes, skeletal muscle cells, and fibroblasts, Rev-erbα influences metabolic gene expression. Changes in expression of metabolic enzymes following Rev-erbα perturbation have been shown to impact several processes such as exercise performance and glucose levels^7^. In excised skeletal muscle, Rev-erbα levels are directly correlated with oxidative capacity, since stabilization of Rev-erbα increases oxygen consumption^8,9^. However, the extent to which Rev-erbα impacts glycolysis, and whether the effect of Rev-erbα on expression of rate limiting glycolytic enzymes is conserved is unclear. Additionally, while datasets detailing transcriptional targets of Rev-erbα in skeletal muscle cells, hepatocytes, and fibroblasts have been developed, there remains a need for mechanistic studies to determine whether these changes have effects on essential cellular functions^10–12^.

This study utilized a mouse embryonic fibroblast (MEF) loss or gain of function model to examine the effect of Rev-erbα disruption on global metabolism. In the absence of Rev-erbα, cells demonstrated an increase in glycolysis, oxidative phosphorylation, proliferation, and vulnerability to oxidative stress. These phenotypes were accompanied by specific upregulation of genes involved in glycolysis and the non-oxidative pentose phosphate pathway (PPP). Upregulated expression of these genes may provide a mechanistic basis for increased metabolism and proliferation resulting from perturbation of Rev-erbα.

## Results

### With Rev-erbα disruption, MEFs demonstrate increased levels of glycolysis and oxidative phosphorylation

**Figure 1:**
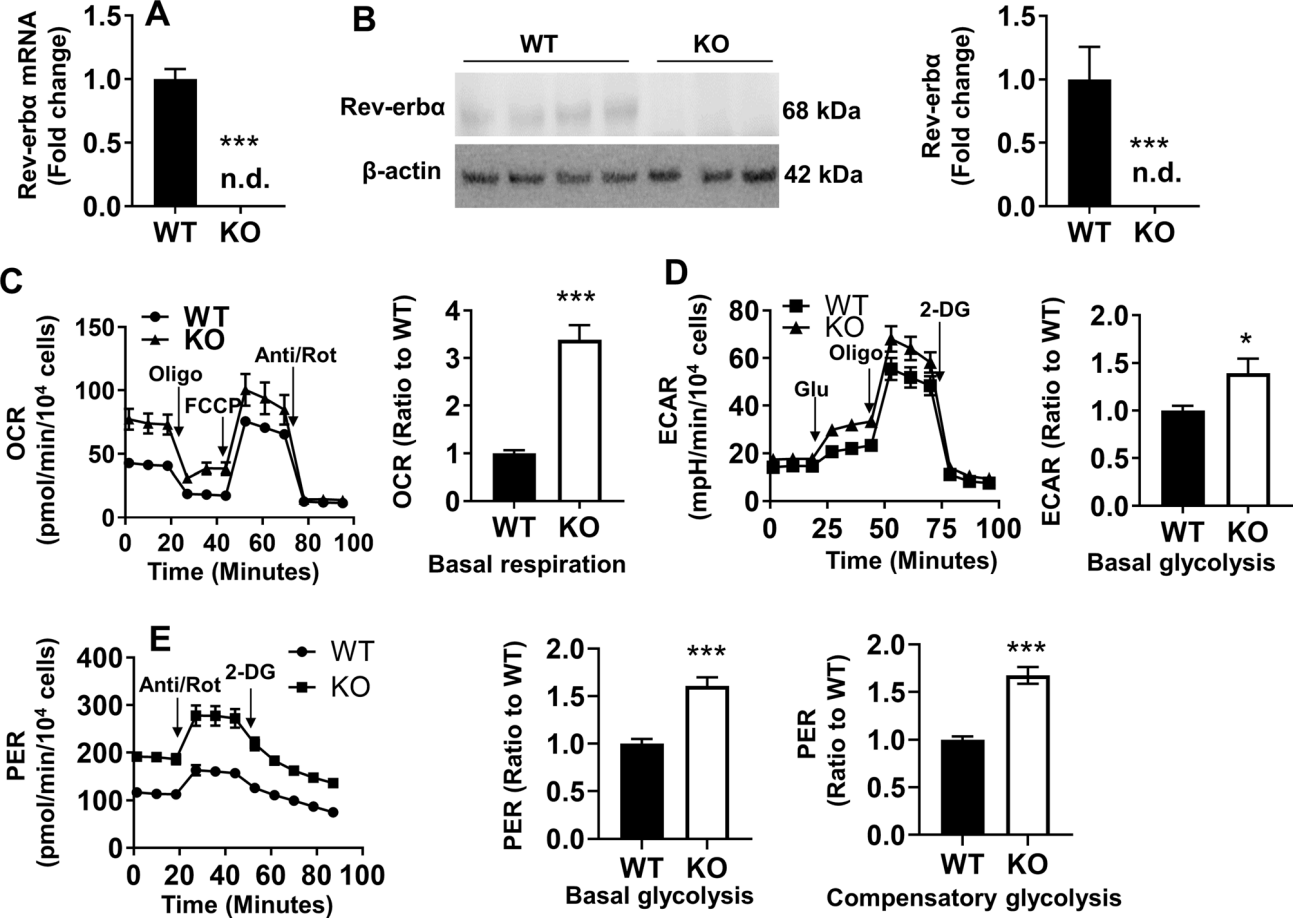
Global metabolism is increased upon loss of Rev-erbα. (**A**) Rev-erbα gene expression in Rev-erbα KO and WT MEFs. n.d. denotes not detected. (**B**) Western blot analysis to measure Rev-erbα protein levels in WT and Rev-erbα KO MEFs. β-actin was used as a loading control. Densitometry analysis of Rev-erbα levels after normalization to β-actin in Rev-erbα KO and WT MEFs. Minimum of n = 5 independent experiments. n.d. denotes not detected. (**C**) Oxygen consumption rate (OCR) trace from the Seahorse mitochondrial stress test after sequential injection of oligomycin (Oligo), FCCP and antimycin A/rotenone (Anti/Rot). Levels of basal respiration calculated in Rev-erbα KO and WT MEFs. (**D**) Extracellular acidification rate (ECAR) trace from glycolysis stress tests after sequential injection of glucose (Glu), oligomycin (Oligo) and 2-deoxy-d-glucose (2-DG). Levels of basal glycolysis calculated in WT and Rev-erbα KO cells. (**E**) Proton efflux rate measured from Seahorse glycolytic rate assays. Levels of basal and compensatory glycolysis were calculated in WT and Rev-erbα KO MEFs. All Seahorse assay traces were normalized to 10,000 cells. Seahorse calculations represented as a ratio to WT group. N = 3–4 independent experiments. Error bars represented as mean ± SEM. *p < 0.05, ***p < 0.001 versus WT.

We previously demonstrated that mouse lung fibroblasts, which expressed a stabilized phosphomimetic form Rev-erbα resistant to proteasomal degradation (SD), are more resistant to nutrient deprivation due to increased oxidative phosphorylation (Oxphos)^13^. Thus, it was hypothesized that Rev-erbα disruption would manifest the reverse phenotype, namely decreased Oxphos. To test this hypothesis, we utilized immortalized Rev-erbα wild type (WT) and knockout (KO) MEFs, as evidenced by Rev-erbα gene expression (Fig. 1A). We also verified that Rev-erbα protein levels were not detected in Rev-erbα KO MEFs (Fig. 1B). Mitochondrial stress tests were then performed using a XF24 Seahorse Analyzer. As shown in Fig. 1C, Rev-erbα KO MEFs demonstrated an increased basal oxygen consumption rate (OCR) compared to WT cells. To examine whether increased oxidative phosphorylation resulted in a bioenergetic shift away from glycolysis, glycolysis stress tests were performed to measure extracellular acidification (ECAR). Surprisingly, higher levels of basal glycolysis were observed in Rev-erbα KO MEFs compared to WT cells (Fig. 1D). To confirm the latter, glycolytic rate assays were also performed to remove the confounding effect of mitochondrial acidification derived from CO_2_ on extracellular acidification. Results were consistent with those of the glycolytic stress test, with Rev-erbα KO MEFs demonstrating increased basal and compensatory glycolysis compared to WT (Fig. 1E). Overall, the increased Oxphos and glycolysis in the MEF KO demonstrate that upon Rev-erbα disruption, there is global metabolic activation.

### Rev-erbα KO MEFs do not exhibit increased mitochondrial mass or mtDNA biogenesis

In a Rev-erbα stabilized cell line, increased oxidative phosphorylation was associated with an increase in mitochondrial area and biogenesis^13^. Additional studies in skeletal muscle also indicate that Rev-erbα overexpression can increase mitochondrial biogenesis^8,9^. To interrogate whether increased Oxphos in the Rev-erbα KO MEFs was associated with an increase in mitochondrial mass, expression of the protein translocase of inner mitochondrial membrane 23 (Timm23) was measured. Rev-erbα KO MEFs did not exhibit increased Timm23 gene expression relative to WT (Supplemental Fig. 1A). Levels of the protein translocase of outer mitochondrial membrane 20 (TOMM20) was also unchanged following Rev-erbα disruption (Supplemental Fig. 1B). In addition, we measured mitochondrial DNA copy number, and quantified the mtDNA/nDNA ratio by qPCR. As shown in Supplemental Fig. 1C and D, the Ct values of 16S, ND1 and HKII as well as ratio of 16S/HKII and ND1/HKII DNA were not altered in Rev-erbα KO MEFs compared to WT cells. These results suggest that the hypermetabolic state of Rev-erbα KO MEFs is not due to increased mitochondrial mass.

### Expression of glycolytic and non-oxidative PPP enzymes is upregulated following Rev-erbα disruption

**Figure 2:**
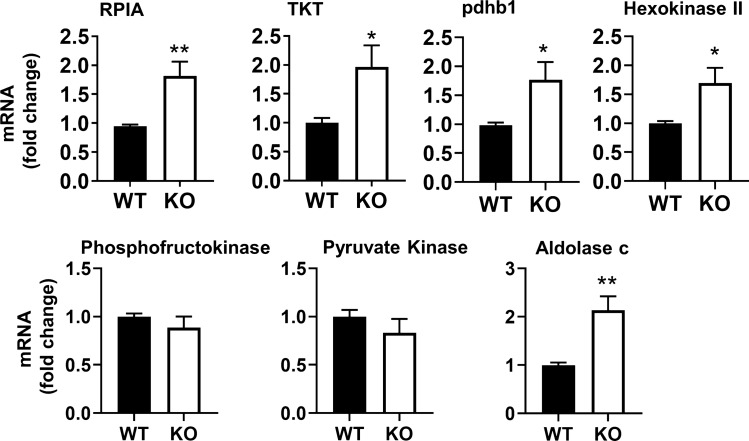
Loss of Rev-erbα leads to upregulation of genes encoding enzymes in the glycolytic and non-oxidative pentose phosphate pathways. Expression of RPIA, TKT, pdhb1, and hexokinase II genes in MEFs cultured in medium containing 10% FBS. n = 5 independent experiments. Error bars represented as mean ± SEM. *p < 0.05, *p < 0.01 versus WT.

To elucidate whether the loss of Rev-erbα mediated transcriptional repression of specific genes regulating glycolysis, a glycolysis gene array was performed. This array included genes encoding enzymes involved in glycolysis, gluconeogenesis, and the PPP (Table 1). In Rev-erbα KO MEFs two of the genes most highly upregulated relative to WT were ribose-5-phosphate isomerase (RPIA) and transketolase (TKT) of the non-oxidative PPP. These are involved in the production of ribose-5 phosphate needed for nucleotide synthesis and the shuttling of metabolic intermediates for glycolysis, respectively^14,15^. Other targets upregulated upon Rev-erbα disruption included aldolase c, an isoform in the canonical glycolysis pathway^16,17^, and pyruvate dehydrogenase subunit b e1 (pdhb1), one of the genes encoding a subunit of the pyruvate dehydrogenase complex^18,19^ (Table 1). Quantitative polymerase chain reaction (qPCR) verified that Rev-erbα KO MEFs had an approximately two-fold increase in each gene (i.e., RPIA, TKT and pdhb1) compared to WT (Fig. 2). Serum starvation can synchronize cells into G0 quiescence. As shown in Supplemental Fig. 2A, serum deprivation significantly increased Rev-erbα protein levels in WT cells. Interestingly, RIPA gene expression was increased whereas TKT transcription was reduced in synchronized cells compared to unsynchronized cells (Supplemental Fig. 2B). As with unsynchronized cells, increased expression of RPIA, TKT, and pdhb1 following Rev-erbα disruption was also observed when the cells were synchronized to control for nutrient availability (Supplemental Fig. 2C). Expression of rate limiting enzymes in glycolysis were additionally measured. In fact, hexokinase II gene expression was elevated in Rev-erbα KO MEFs relative to WT cells, and this was maintained following synchronization (Fig. 2; Supplemental Fig. 2B). Overall, these data demonstrate a cell-cycle specific Rev-erbα expression and its repressive role in the non-oxidative PPP and in glycolysis.

**Table 1:** Changes in gene expression in Rev-erbα KO MEFs relative to WT by a glycolysis gene array.

Genes	Fold change	Full name of genes
Aldob	− 3.50	Aldolase B, fructose-bisphosphate
Aldoc	2.11	Aldolase C, fructose-bisphosphate
Cs	1.67	Citrate synthase
Dlat	1.5	Dihydrolipoamide S-acetyltransferase (E2 component of pyruvate dehydrogenase complex)
Dlst	− 8.54	Dihydrolipoamide S-succinyltransferase (E2 component of 2-oxo-glutarate complex)
Eno1	− 7.20	Enolase 1, alpha non-neuron
Eno2	− 1.66	Enolase 2, gamma neuronal
Fbp1	− 1.31	Fructose bisphosphatase 1
G6pc3	− 5.12	Glucose 6 phosphatase, catalytic, 3
G6pdx	− 1.36	Glucose-6-phosphate dehydrogenase X-linked
Galm	− 1.19	Galactose mutarotase
Gpi1	1.39	Glucose phosphate isomerase 1
Gys1	− 1.76	Glycogen synthase 1, muscle
Gys2	− 1.62	Glycogen synthase 2
Hk2	− 1.76	Hexokinase 2
Hk3	− 1.72	Hexokinase 3
Idh3a	1.02	Isocitrate dehydrogenase 3 (NAD +) alpha
Idh3b	− 2.00	Isocitrate dehydrogenase 3 (NAD +) beta
Idh3g	− 1.36	Isocitrate dehydrogenase 3 (NAD +), gamma
Mdh1	1.1	Malate dehydrogenase 1, NAD (soluble)
Ogdh	1.75	Oxoglutarate dehydrogenase (lipoamide)
Pcx	1.25	Pyruvate carboxylase
Pdha1	− 1.9	Pyruvate dehydrogenase E1 alpha 1
Pdhb	4.82	Pyruvate dehydrogenase (lipoamide) beta
Pdk1	1.79	Pyruvate dehydrogenase kinase, isoenzyme 1
Pdk2	− 16.05	Pyruvate dehydrogenase kinase, isoenzyme 2
Pdk3	− 1.77	Pyruvate dehydrogenase kinase, isoenzyme 3
Pdp2	− 1.12	Pyruvate dehyrogenase phosphatase catalytic subunit 2
Pgam2	− 2.66	Phosphoglycerate mutase 2
Pgk2	− 4.51	Phosphoglycerate kinase 2
Phkb	− 3.58	Phosphorylase kinase beta
Phkg1	− 2.15	Phosphorylase kinase gamma 1
Prps1	1.55	Phosphoribosyl pyrophosphate synthetase 1
Prps1l1	− 1.36	Phosphoribosyl pyrophosphate synthetase 1-like 1
Prps2	1.61	Phosphoribosyl pyrophosphate synthetase 2
Pygl	170.28	Liver glycogen phosphorylase
Pygm	− 2.18	Muscle glycogen phosphorylase
Rbks	1.78	Ribokinase
Rpe	− 1.9	Ribulose-5-phosphate-3-epimerase
Rpia	1.96	Ribose 5-phosphate isomerase A
Sdhb	1.39	Succinate dehydrogenase complex, subunit B, iron sulfur (Ip)
Sucla2	1.00	Succinate-Coenzyme A ligase, ADP-forming, beta subunit
Suclg2	− 2.11	Succinate-Coenzyme A ligase, GDP-forming, beta subunit
Taldo1	− 1.79	Transaldolase 1
Tkt	2.04	Transketolase

### Inverse effects of Rev-erbα disruption and stabilization on glycolysis and non-oxidative PPP expression

**Figure 3:**
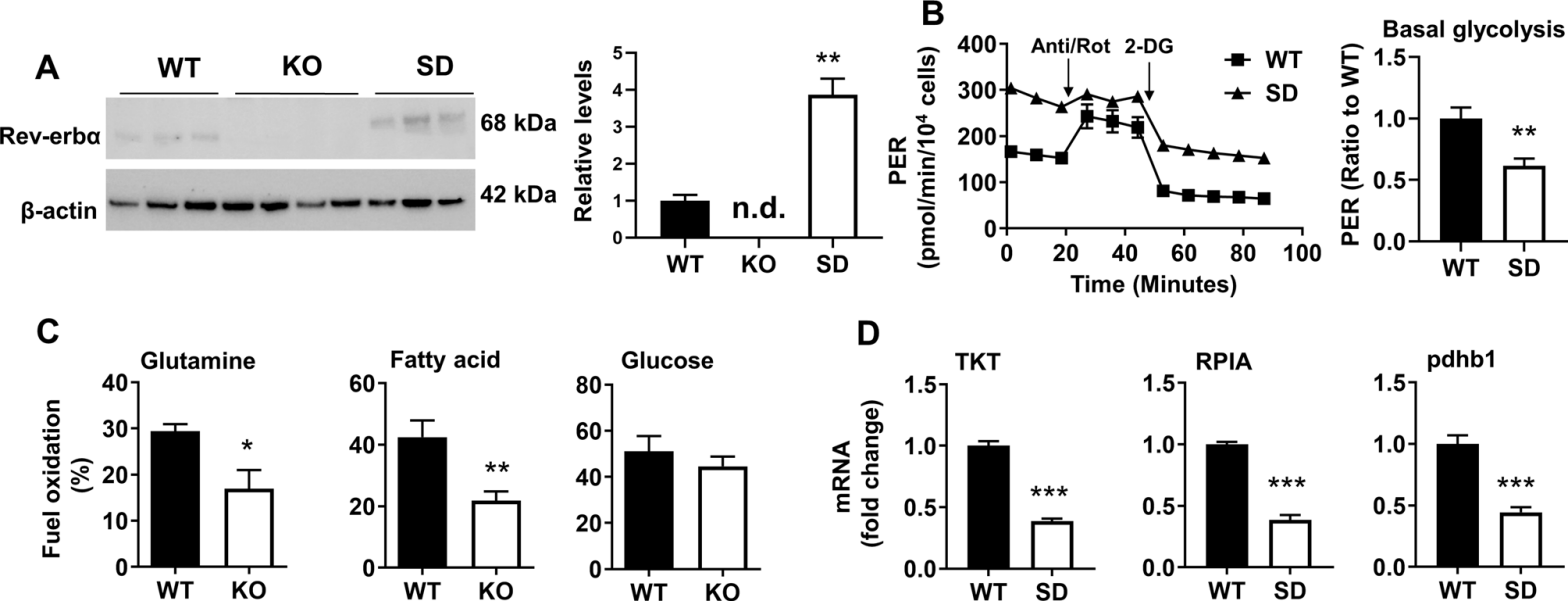
Rev-erbα stabilization reduces levels of glycolysis as well as glycolytic and PPP enzyme expression. (**A**) Western Blot analysis to measure Rev-erbα protein levels in Rev-erbα KO, SD, and WT cells. β-Actin was used as a loading control. Densitometry analysis of Rev-erbα levels in Rev-erbα KO, SD, and WT cells. n.d. denotes not detected. (**B**) Proton efflux rate trace for WT and Rev-erbα SD MEFs normalized to cell number. Anti/Rot: antimycin A/rotenone. Levels of basal and compensatory glycolysis represented as ratios to WT. (**C**) Fuel dependency analysis in WT and Rev-erbα KO MEFs. Glutamine, fatty acid, and glucose dependency are denoted as a percentage of total fuel oxidation. (**D**) Expression of TKT, RPIA, and pdhb1 genes in WT and Rev-erbα SD cells. n = 3 independent experiments. Error bars represented as mean ± SEM. *p < 0.05, **p < 0.01, ***p < 0.001 versus WT.

Fibroblasts expressing a phosphomimic form of Rev-erbα (SD) to protect Rev-erbα from proteasomal degradation and extent protein half-life, were utilized^20^. These cells demonstrated a threefold increase in Rev-erbα protein levels relative to WT (Fig. 3A) and reduced levels of basal glycolysis as expected (Fig. 3B). The phosphomimic form of Rev-erbα migrated more slowly on the SDS gel, most likely owing to reduced interactions with sodium dodecyl sulfate or negatively charged aspartate^21,22^. However, these cells maintained a high proton efflux following administration of 2-deoxy-glucose (2-DG) compared to WT cells. This led to reduction in basal glycolysis in SD cells compared to WT cells (Fig. 3B).

Although Oxphos was increased, fuel flexibility tests revealed that following Rev-erbα disruption, there was a reduced dependence on glutamine and fatty acids for energy production, while glucose utilization remained consistent (Fig. 3C). These results suggest anaplerotic reactions may contribute to increased Oxphos in Rev-erbα KO cells. Expression of RPIA and TKT was reduced in Rev-erbα SD cells relative to WT (Fig. 3D), suggesting that this inverse relationship may extend to non-oxidative PPP enzymes. Expression of the Pdhb1 gene was additionally reduced in the Rev-erbα SD cells (Fig. 3D), which confirmed negative regulation of this gene by Rev-erbα.

### Rev-erbα does not regulate expression of target genes through BMAL1

To determine whether Rev-erbα modulates metabolic gene expression through BMAL1, we first determined expression of the Arntl gene in Rev-erbα KO cells. As shown in Supplemental Fig. 3A, Rev-erbα KO MEFs exhibited increased expression of Arntl relative to WT cells. The promoter of RPIA contains E-Box elements which could potentially bind BMAL1^1–3^, allowing for an indirect mechanism for the observed increased RPIA expression in Rev-erbα KO MEFs. To test this hypothesis, Arntl expression was partially ablated using small interfering RNAs (Supplemental Fig. 3B). This only led to a modest decrease in RPIA gene expression in Rev-erbα KO cells (Supplemental Fig. 3B). Interestingly, upon robust overexpression of Arntl in WT cells, expression of hexokinase II, RPIA, TKT, and pdhb1 were unchanged (Supplemental Fig. 3C and D). Thus, Rev-erbα is unlikely to indirectly control the expression of these glycolysis and PPP enzymes through BMAL1.

### Cell numbers and proliferation are increased following Rev-erbα disruption in MEFs

**Figure 4:**
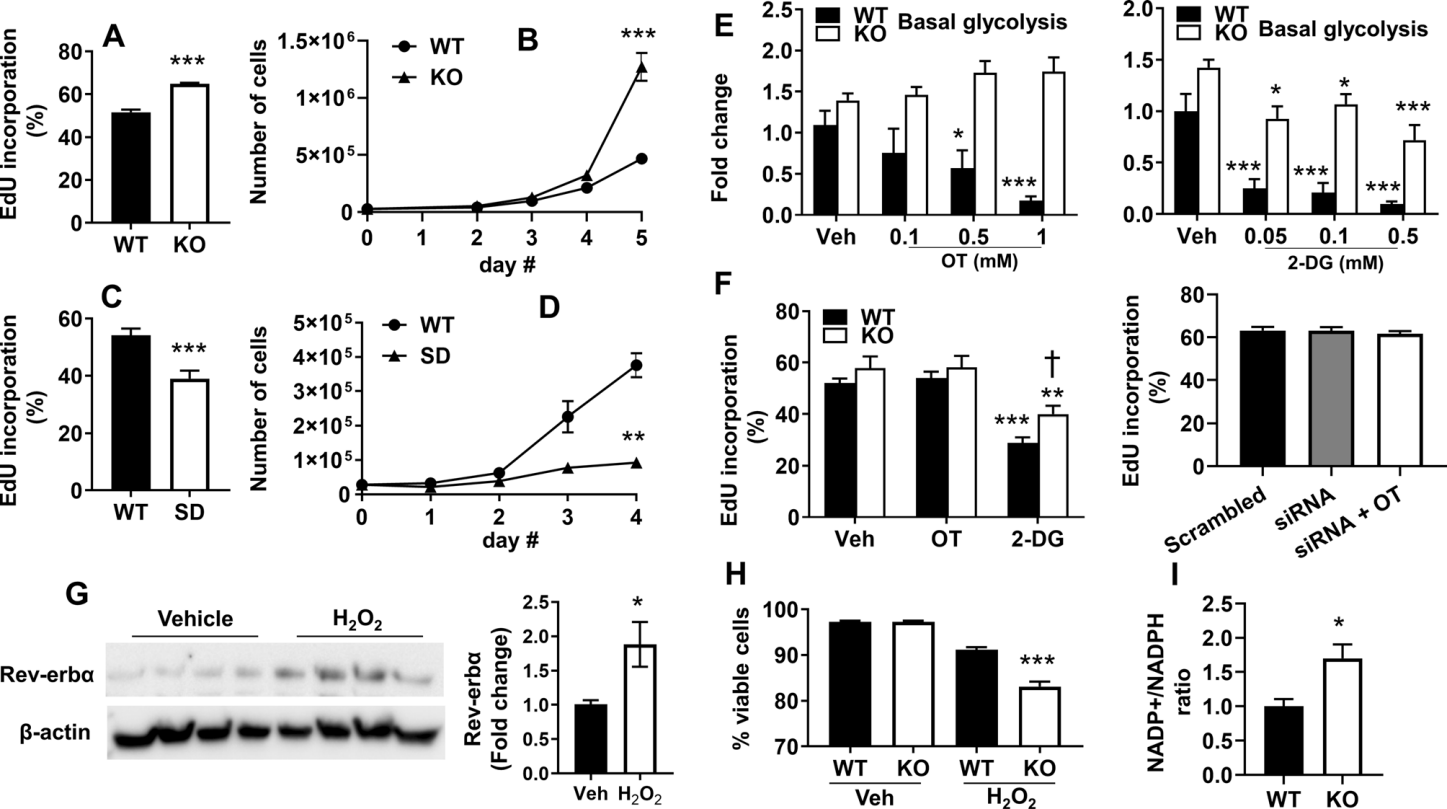
Loss of Rev-erbα increases proliferation, cell growth, and vulnerability to oxidative stress. (**A**) EdU incorporation assays performed with WT and Rev-erbα KO MEFs. (**B**) A growth curve of WT and Rev-erbα KO MEFs with counts over a 5-day span at daily intervals. (**C**) Proliferation assays performed with WT and Rev-erbα SD MEFs. (**D**) A growth curve of WT and SD MEFs with counts over a 4-day span at daily intervals (right panel). (**E**) Basal glycolysis of Rev-erbα KO and WT MEFs treated with oxythiamine (OT) or 2-deoxy-glucose (2-DG) for 24 h. Glycolytic levels represented as a ratio to WT/vehicle group. (**F**) Proliferation assays performed with Rev-erbα KO and WT MEFs following treatment with 0.5 mM of OT and 2-DG, or transfection with RPIA siRNA. (**G**) Rev-erbα protein was measured by Western blot in WT MEFs treated with H_2_O_2_ (500 µM) for 24 h. (**H**) Viability of WT and Rev-erbα KO MEFs following 24 h treatment with 500 µM H_2_O_2_ measured by trypan blue exclusion. (**I**) Ratio of NADP^+^/NADPH in WT and Rev-erbα KO MEFs. n = 3 independent experiments. Error bars represented as mean ± SEM. *p < 0.05, **p < 0.01, ***p < 0.001 versus WT (**A**–**D**, **H** and **I**), vehicle (**E**–**G**) by unpaired *t *test; ^†^*p* < 0.05 versus WT/2-DG group.

The enzymes RPIA and TKT are known to be important for the synthesis of ribose-5-phosphate, a precursor for nucleotide biosynthesis^23–25^. It may be possible that increased nucleotide biosynthesis and metabolism in Rev-erbα KO MEFs facilitate the demands of increased proliferation. To determine if Rev-erbα impacts cell proliferation, a 5-ethynyl-2′-deoxyuridine (EdU) incorporation assay was performed. Rev-erbα KO MEFs demonstrated increased proliferation relative to WT (Fig. 4A). To determine if the increased proliferation manifested as increased cell numbers, a 5-day growth curve as performed. Rev-erbα KO MEFs demonstrated dramatically increased cell numbers by day 5 (Fig. 4B). Conversely, proliferation and cell numbers were reduced in the Rev-erbα SD MEFs compared to WT (Fig. 4C, D).

To determine whether increased proliferation was dependent on the PPP based nucleotide biosynthesis or glycolysis, Rev-erbα KO and WT cells were treated with the TKT inhibitor oxythiamine^26–28^ or the hexokinase inhibitor 2-DG^29,30^. Glycolytic rate assays demonstrated that both 2-DG and oxythiamine reduced glycolysis in WT cells (Fig. 4E). These effects were attenuated in Rev-erbα KO cells (Fig. 4E). Furthermore, treatment with 2-DG, but not oxythiamine, reduced cell proliferation in both Rev-erbα KO and WT MEFs (Fig. 4F). Similarly, EdU incorporation was not altered in Rev-erbα KO transfected with RPIA siRNA transfection in the presence or absence of oxythiamine compared to the scrambled siRNA group (Fig. 4F). Compared to WT cells, 2-DG-mediated reduction of EdU incorporation was less in Rev-erbα KO cells (Fig. 4F). These results demonstrate that increased glycolysis contributes to the hyperproliferative phenotype of Rev-erbα KO MEFs compared to WT cells.

### Loss of Rev-erbα increases vulnerability to oxidative stress

Our previous studies showed that stabilization of Rev-erbα in MEFs is protective against hydrogen peroxide (H_2_O_2_)-induced oxidative stress and that oxidative stress can influence Rev-erbα transcriptional repression^13,31^. At baseline, Rev-erbα KO and WT MEFs exhibited comparable levels of oxidative stress, as measured by 5,5-dimethyl-1-pyrroline N-oxide (DMPO) protein adducts^32,33^ (Supplemental Fig. 4A). Additionally, expression of heme oxygenase 1 (HMOX1) and manganese superoxide dismutase 2 (SOD2) genes, encoding enzymes known to upregulated in response to oxidative stress, were unchanged upon Rev-erbα disruption^34–36^ (Supplemental Fig. 4B). Furthermore, SOD2 protein levels were also unchanged in Rev-erbα KO MEFs (Supplemental Fig. 4C). Although 24 h of H_2_O_2_ incubation increased Rev-erbα protein levels in WT cells, Rev-erbα KO MEFs exhibited reduced viability relative to WT cells (Fig. 4G, H). Rev-erbα stabilization has been shown to upregulate HMOX1 and SOD2 gene expression in response to H_2_O_2_ incubation^13^. This finding led us to hypothesize that expression of these enzymes would be reduced following Rev-erbα disruption in response to oxidative stress. Surprisingly, HMOX1 expression was increased in H_2_O_2_-treated KO MEFs, whereas SOD2 was unchanged, compared to WT cells (Supplemental Fig. 4D). This suggests that, in contrast to the Rev-erbα SD, where increased expression of antioxidant enzymes increased cell viability^13^, increased expression of HO-1 does not influence viability of Rev-erbα KO.

Although Rev-erbα KO MEFs exhibited increased expression of TKT and RPIA, inhibition of these enzymes did not affect proliferation (Fig. 4F, right panel). The non-oxidative PPP is downstream of the oxidative PPP, which produces nicotinamide adenine dinucleotide phosphate (NADPH) necessary for the reduction of the key antioxidant enzyme glutathione. Rev-erbα KO MEFs exhibited a higher ratio of NADP^+^/NADPH compared to WT cells (Fig. 4I), suggesting that perturbation of the PPP following Rev-erbα disruption may underlie reduced viability to oxidative stress.

## Discussion

This study demonstrates that Rev-erbα regulates glycolysis by specific upregulation of enzymes in the PPP as well hexokinase, a rate-limiting enzyme in glycolysis. This is associated with increased proliferation and growth in MEFs. Several reports have linked Rev-erbα to oxidative phosphorylation^9,13,37^. Here we show that Rev-erbα KO MEFs had increased oxidative phosphorylation relative to WT cells. Paradoxically, Rev-erbα SD MEFs also demonstrated an increase in oxidative phosphorylation. Previous studies indicate overexpression of Rev-erbα in fibroblasts and skeletal muscle cells leads to an increase in mitochondrial mass and biogenesis relative to WT^8,9,13^, we did not see any change levels of mitochondrial mass and mtDNA content, suggesting that loss of Rev-erbα does not result in changes in mitochondrial mass or numbers. This difference in phenotype may explain the paradoxical relationship between Rev-erbα levels and oxidative phosphorylation. We noticed that HKII mRNA was increased in KO cells, there were no changes of HKII DNA copy number in these cells compared to WT cells. Further study is required to determine the mechanisms underlying discrepancies between DNA copy number and mRNA levels in Rev-erbα KO cells. It remains elusive whether Rev-erbα deletion upregulates metabolism and proliferation in other types of cells, including epithelial cells, muscle cells and hepatocytes.

While Rev-erbα levels can govern the expression of genes involved in hepatocyte cholesterol metabolism^38,39^, gluconeogenesis^40–42^, and the immune response^43–45^, studies detailing the effect of Rev-erbα levels on expression of glycolytic enzymes have been less conclusive, with different patterns of expression of rate limiting enzymes evident in skeletal muscle cells, fibroblasts, and hepatocytes^7,13^. Additionally, little research has been done to interrogate the effect of Rev-erbα on the overall output of the glycolytic pathway. Rev-erbα disruption in MEFs led to specific upregulation of hexokinase, TKT, pdhb1, and aldolase c in unsynchronized cells. The presence of an E-Box element in the promoter of RPIA gene suggested there may be an indirect mechanism of transcriptional regulation through BMAL1 which known to have its expression repressed by Rev-erbα^1–3^. Additionally, recent evidence has suggested that expression of aldolase c may be driven in part through BMAL1^46^. However, we found that while expression RPIA, TKT, and pdhb1 demonstrated an inverse relationship with Rev-erbα levels, overexpression of BMAL1 in WT MEFs did not lead to changes in expression of these genes. This suggest that BMAL1 may not be involved in upregulation of these genes in Rev-erbα KO cells. Further experiments with BMAL1 deletion or overexpression in both Rev-erbα KO and WT MEFs in the same circadian phase would reveal whether BMAL1 mediates hypermetabolism and hyperproliferation in Rev-erbα KO cells.

It is possible that Rev-erbα exerts control of glycolysis and the PPP through a mechanism that is divergent from the traditional circadian clock. Amongst the more notable studies providing evidence for this was the finding that Rev-erbα can interact with the liver lineage transcription factor hepatocyte nuclear factor 6 to repress gene expression in hepatocytes^11^. A recent study demonstrates that Rev-erbα controls more extensive transcription in the liver under conditions of metabolic perturbation such as mis-timed feeding^47^. This suggests that the repressive action of Rev-erbα does not drive basal rhythmicity in metabolic activity but serves to buffer against metabolic perturbation in the liver. Recent evidence in a Rev-erb α/β double KO MEF cell line found enrichment for binding of the hepatocyte nuclear factor 1 and nuclear transcription factor 1 in the promoters of genes that halted circadian oscillation following Rev-erb disruption. While our experiments in synchronized MEFs suggest that RPIA, TKT, pdhb1, and hexokinase II may be under circadian control, these genes were not found to be oscillating in double KO MEFs^12^. Future work is required to determine the extent to which Rev-erbα mechanistically influences the expression of these metabolic genes and the circadian genes, including Cry, Per and Nfil3, in a clock-dependent or independent manner. Cells become quiescent (entering the G0 phase) during serum deprivation. Interestingly, Rev-erbα was increased in synchronized MEFs with serum deprivation. This suggests that Rev-erbα is regulated in a cell-cycle dependent manner. Serum shock is able to phase-lock the cells and alters Rev-erbα expression in fibroblasts^48^. Future study using serum shock (i.e., treating cells with high concentrations of serum) will further validate the role of Rev-erbα in modulating glycolysis, the PPP and proliferation.

Glycolysis and the PPP make important contributions toward cellular proliferation. Cancer cells are known to increase aerobic glycolysis via the Warburg effect to drive uncontrolled proliferation^49–51^. A number of studies have suggested that Rev-erbα may be a viable therapeutic target for the treatment of breast, colon, brain, and gastric cancers^52–57^. Studies in cancerous and non-cancerous cell lines have demonstrated that incubation with the synthetic Rev-erbα agonists SR9009 and GSK4112 can impede proliferation^52,54,58^. However, recent findings have indicated that SR9009 may have nonspecific effects on cell numbers and delay cell cycle progression in the absence of Rev-erbα^37^. The findings of the present study indicate that Rev-erbα KO and SD cells have increased and decrease proliferation relative to WT cells, respectively, which corroborates with previous research suggesting that Rev-erbα can regulate proliferation.

We show that Rev-erbα KO MEFs displayed increased glycolysis and proliferation resembling the Warburg effect. This hyperproliferative phenotype may be driven by upregulation of the non-oxidative PPP. The PPP is known to be the main source of nucleotides needed for DNA replication during cell division, with the non-oxidative arm of the PPP being predominantly involved in the production of ribose-5 via RPIA, which directly catalyzes the production of ribose-5. Expression of these enzymes is important to proliferation of cancer cell lines. In breast cancer cell lines, silencing of TKT led to cell cycle arrest coupled with metabolic flux towards glycolysis away from nucleotide biosynthesis^23^. Additionally, in an in vitro model of colon cancer cells, silencing of RPIA led to reduced nucleotide incorporation and cell survival^24^. Although we could demonstrate a clear inverse relationship between Rev-erbα levels and non-oxidative PPP gene expression, we were unable to observe any associated change in cell proliferation following inhibition of TKT or ablation of RPIA.

In contrast, inhibition of hexokinase II reduced proliferation. Elevated expression of hexokinase II is known to be a hallmark of several cancer cell lines providing needed metabolic intermediates for glycolysis, oxidative phosphorylation, and nucleotide biosynthesis^59–61^. In WT MEFs, inhibition of hexokinase II using 2-DG led to a reduction in nucleoside incorporation. These effects were attenuated in Rev-erbα KO cells, which may be due to high glycolysis in these cells. Increased glycolysis, characteristic of the Warburg effect, has also been shown to be driven by phosphofructokinase and pyruvate kinase, which mediate additional rate limiting steps in glycolysis^62–65^. Expression of these enzymes was unchanged following Rev-erbα disruption, suggesting hexokinase alone drove increased glycolysis and proliferation in this background. This insight provides new evidence that loss of transcriptional repression not associated with oncogenesis may drive a hypermetabolic, hyperproliferative phenotype.

While perturbation of the non-oxidative PPP did not affect glycolysis or proliferation, it is possible that Rev-erbα-mediated changes in non-oxidative PPP expression may affect antioxidant defense. Oxidative stress has been previously demonstrated to drive Rev-erbα transcription through nuclear erythroid factor 2^31^. Conversely, to date, the effect of Rev-erbα on the oxidative stress response has been controversial. Rev-erbα stabilization provides protection in fibroblasts against hydrogen peroxide stress through induction of HO-1, SOD2, and catalase^13^. However, in a breast cancer cell line, Rev-erbα inhibits Poly [ADP-ribose] polymerase 1 leading to an increase in DNA damage and cell death^56^. The increase in Rev-erbα in WT cells treated with H_2_O_2_ may be compensatory and protective against cell death. Rev-erbα KO MEFs under oxidative stress exhibited reduced viability that was not accompanied by a reduction in HO-1 or SOD2 expression. This suggests that the diminished cellular pool of NADPH following Rev-erbα disruption contributes more to increased oxidative vulnerability than antioxidant enzymatic expression or DNA damage. The possibility that diminished NADPH contributes to oxidation of the important antioxidant GSH in the Rev-erbα KO cells remains to be determined. The PPP is thought to contribute to the regeneration of the reducing agent NADPH. Although Rev-erbα KO cells displayed increased glycolysis and PPP, levels of NADPH were reduced in these cells. We surmise that NADPH may be consumed for macromolecule (e.g. fatty acids) synthesis to increase proliferation in the Rev-erbα KO cells. Further studies using a metabolic flux assay are required to determine whether Rev-erbα deletion causes a metabolic switch between glycolysis and the PPP.

In summary, disruption of Rev-erbα results in increased glycolysis, proliferation, and cell growth. These phenotypic changes are driven by specific changes in hexokinase II expression that is inversely proportional to Rev-erbα levels. Furthermore, specific enzymes of the PPP are also increased in response to Rev-erb disruption, leading to diminished pools of NADPH and subsequent reduced protection against oxidative stress.

## Experimental procedures

### Cell culture, synchronization and treatment

MEFs were used as an in vitro model for this study. Fibroblasts were prepared from E13.5 WT and Rev-erbα Global Knockout Embryos (gift of Mitch Lazar, University of Pennsylvania, Philadelphia, PA, USA) using standard protocols. The cells were transfected with an SV40 plasmid for immortalization and maintained in DMEM (Caisson Laboratories, Smithfield, UT, USA) supplemented with 10% bovine serum, penicillin/streptomycin, and glutamine. Cells were maintained at 37 °C under 5% CO_2_ condition. Synchronized MEF cells were seeded, allowed to attach for 2 h, after which they were cultured in serum free DMEM for 24 h. Cells were then cultured in serum containing DMEM for an additional 48 h. Serum starvation followed by replacement with serum containing medium aligns the cells in G0 quiescence. For transketolase inhibition experiments, cells were treated with oxythiamine (Sigma Aldrich, catalog#: 614-05-1) for 24 h. Cells were treated and collected for endpoint measurements at similar times of day for consistency.

### Mitochondrial stress test

Parameters of oxidative phosphorylation were measured using the mitochondrial stress test on the Seahorse XF24 Bioanalyzer (Agilent, Santa Clara, CA, USA) as described previously^66^. Cells were seeded in Seahorse assay plates 24 h in air (21% O_2_/5% CO_2_) prior to assay. Cells were inspected the day of the assay to ensure uniform confluence and one well of each genotype was sacrificed for cell counting. OCR was normalized to 10,000 cells per well and all parameters were normalized to the average of WT group as a ratio. Basal respiration is defined as the initial level of oxygen consumption rate, and maximal respiration is the level of oxygen consumption rate measured following FCCP injection.

### Glycolysis stress test

Glycolysis stress tests were performed as an indicator of glycolytic levels as described previously^67^. MEF cells were seeded and incubated overnight as described for mitochondrial stress tests. ECAR was used as an index of glycolysis with initial levels normalized to cell number for each genotype. Basal glycolysis was calculated based on the differences in ECAR after glucose injection, and the data are expressed as a relative ratio of 1 normalized to WT.

### Glycolytic rate assay

MEF cells were seeded and incubated overnight as described for mitochondrial stress tests. Proton efflux rate response following injections of the electron transport chain inhibitors rotenone/antimycin A and the glucose analog 2-DG was measured to determine basal and compensatory glycolysis as described previously^67^. Data were analyzed using the Seahorse Glycolytic Rate assay report generator, and basal glycolysis was calculated based on the differences in PER after 2-DG injection. The data were normalized to cell number for each genotype, and converted to a ratio of 1 with respect to WT.

### Fuel flexibility assay

To determine dependency on glutamine, fatty acids, and glucose, fuel flexibility assays were performed^66^. MEF cells were seeded and incubated overnight as described for the mitochondrial stress test. The drop in basal consumption following injections of BEPTES, UK5099, and etomoxir were quantified as a percentage of basal respiration for glutamine, glucose, and fatty acids, respectively.

### Glycolysis gene array

A Qiagen Glucose Metabolism RT^2^ Profiler PCR Array (Qiagen, Hilden, Germany) was used to determine expression levels of genes involved in glycolysis, gluconeogenesis, or the pentose phosphate pathway. Cells were pooled from 3 samples in each condition. RNA was isolated using an RNeasy prep kit that was also from Qiagen. Expression of microchip array targets was measured using a GeneChip Instrument (Affymetrix, Santa Clara, CA, USA).

### Quantitative RT-PCR

Quantitative RT-PCR was used to measure relative expression of metabolic enzymes. Relative expression of all mRNA targets was tested using the TaqMan gene expression system (Applied Biosystems, Foster City, CA, USA). Total RNA was extracted using the TRIzol Plus RNA Purification System (Invitrogen, Carlsbad, CA, USA) or a Qiagen RNeasy Kit (Qiagen, Hilden, Germany). A NanoDrop One (Thermo Scientific, Waltham, MA, USA) instrument was used to test the quantity and concentration of RNA. For *arntl* silencing and overexpression, cell lysis was performed in plate with a Qiagen RNeasy kit (Germantown, MD) used for downstream RNA processing according to manufacturer protocols. cDNA was synthesized using the TaqMan Reverse Transcription System and run on an Applied Biosystems 7300 Real-Time PCR instrument. TaqMan probes were as follows: nr1d1 (Mm00520711), RPIA (Mm00485790), transketolase (Mm00447559), arntl (Mm00500226), pyruvate kinase muscle isoform (m1/m2) (Mm00834102), pyruvate kinase liver/red blood cell isoform (Mm00443090), phosphofructokinase (Mm01309576), hexokinase I (Mm00439344), hexokinase II (Mm00443385), aldolase c (Mm01298116), and pdhb (Mm00499323). Samples were denatured at 95 °C for 10 min, followed by 40 cycles of denaturing at 95° for 15 s followed by extension at 60 °C for 1 min.

### Mitochondrial DNA measurement

DNA was extracted using an organic phenol extraction buffer (phenol:chloroform at a ratio of 5:3), and precipitated with absolute alcohol cooled to − 20 °C containing sodium acetate (0.3 M) as described previously^66^. DNA (40 ng) was used for quantitative PCR to measure the relative copy number of mtDNA (16S and ND1) and nDNA (hexokinase 2, HK2). The primers are shown as follows, 16S: fwd: 5′-CCGCAAGGGAAAGATGAAAGAC-3′; rev: 5′-TCGTTTGGTTTCGGGGTTTC-3′, ND1: fwd: 5′-CTAGCAGAAACAAACCGGGC-3′; rev: 5′-CCGGCTGCGTATTCTACGTT-3′, HK2: fwd: 5′-GCCAGCCTCTCCTGATTTTAGTGT-3′; rev: 5′-GGGAACACAAAAGACCTCTTCTGG-3′. PCR reaction mixtures included SYBR Green Supermix (Cat#:172-5271, Bio-Rad), primer master mixtures, DNA (20 ng/µl) and free water. The reactions were performed at 50 °C for 2 min, and following 40 cycles at 98 °C for 10 min, 98 °C for 15 s, 60 °C for 60 s. Analysis of mtDNA/nDNA ratio was be calculated by the classical 2^-ΔΔCt^ method used for qPCR analysis.

### Gene silencing and overexpression

Lipid mediated siRNA transfection was used to knock down *arntl* expression. For *anrtl* silencing, pooled *arntl* Qiagen flexitube siRNAs (catalog # 1027416) were transfected into KO MEFs using Thermo Fisher RNAiMAX reagent with transfection performed per manufacturer instructions. Thermo Fisher Ambion siRNA (catalog # AM16708) was used for ribose-5-phosphate isomerase (RPIA) silencing. *Arntl* was overexpressed in WT MEFs using an Origene pCMV6 vector plasmid (catalog # MR209553) using Thermo Fisher Lipofectamine 3000 reagent. RNA was harvested 24 h post transfection.

### EdU incorporation assay

Nucleoside incorporation assays were performed as an index of proliferation as described previously^67^. Cells were seeded 24 h prior to assay start time. EdU is a thymidine analog that incorporated into cell during DNA synthesis in S phase of the cell cycle to quantify the proportion of cell undergoing activate proliferation. Cells were then treated with 3 µM EdU provided by the Click-iT EdU proliferation assay (Invitrogen, Carlsbad, CA, USA). Cells prepared according to manufacturer’s protocol. Flow Cytometry was then performed using a BD FACSAria IIIu at the Brown Flow Cytometry core (Providence, RI, USA), with cells from the main population registering as Alexa Fluor 488 positive deemed proliferative. A minimum of 20,000 events were recorded per sample. For non-oxidative PPP experiments, cells were treated with oxythiamine or transfected with RPIA siRNA 24 h prior to EdU treatment.

### Growth curves

For these experiments, 28,000 MEFs were seeded in 6 cm^2^ plates for the WT and Rev-erbα SD genotypes. For Rev-erbα KO cells, we seeded 14,000 cells in a plate so as to avoid over confluence at the end of the experiment since these cells grow significantly faster than WT. Three plates of MEFs were trypinisized and counted on a hemocytometer using trypan blue reagent over a period of four to five days, with live cell counts averaged and standard error of the mean calculated.

### Immunoblotting

Western blotting was used to measure relative protein levels. Total protein was isolated from Rev-erbα WT, KO, and SD MEFs. Lysate was mixed with β-mercapaethanol, boiled, run on a 4–12% Sodium Dodecyl Sulfate Polyacrylamide Gel (Invitrogen, Carlsbad, CA, USA), and transferred to a PVDF membrane. Membranes were incubated with the following primary antibodies and dilutions: Rev-erbα (#13,418, Cell Signaling Technologies, Danvers, MA monoclonal rabbit antibody, 1:1000), TOMM20 (#42406S Cell Signaling Technologies, Danvers, MA, USA, 1:5000), DMPO (gift of Ronald Mason, National Institute for Environmental Health Sciences, Research Triangle Park, NC, USA), SOD2 (#06-984, Upstate EMD Millipore, Burlington, MA, USA, 1:1000). β-actin (#ab8227, abcam, Cambridge, MA, USA) and calnexin (#ADI-SPA-860-F, Enzo, Farmingdale, NY, USA) were used as loading controls.

### Cell viability assays and H_2_O_2_ treatment

To test for a difference in baseline viability, MEFs were seeded in 6 cm^2^ plates. Total cells were collected for up to 5 days post seeding. Cells were mixed with trypan blue. Cells excluding trypan blue were denoted as live with the percentage of live cells divided total used to calculate viability. Cells treated with H_2_O_2_ received treatment for 24 h at a 500 µM concentration.

### NADP^+^/NADPH ratio determination

Reagents from Abcam (catalog # ab65349) were used according to manufacturer protocols as described previously^67^. Three million WT and Rev-erbα KO cells were utilized for each biological measurement. DNA was sheared via syringing prior to NADP^+^ decomposition. Optical plates were read at 1 h following addition of assay developer. For individual total species and NADPH quantities, assay readings were normalized to protein amounts quantified via a BCA assay.

### Statistical analysis

Experiments were repeated biologically three times, with three technical triplicates for quantitative PCR and functional assays. Seahorse assays contained up to nine technical replicates each. Error bars are reported as the standard error of the mean. Graph Pad Prism 9 software was used for t-test and ANOVA analysis. The unpaired *t* test was used for detecting statistical significance of the differences between means of two groups if the data are normally distributed. Welch-corrected *t* test was used if the data are not normally distributed. The statistical significance of the differences among multiple groups was evaluated by using one-way ANOVA for overall significance, followed by Tukey–Kramer test. Statistical significance was considered when the *p* value was less than 0.05.

## Supplementary Information

Supplementary Information.

## Abbreviations

2DG: 2-Deoxy-glucose
Aldoc: Aldolase C
DMPO: 5,5-Dimethyl-1-pyrroline N-oxide
HO-1: Heme oxygenase 1
KO: Knockout
MEF: Mouse embryonic fibroblast
NADPH: Nicotinamide adenine dinucleotide phosphate
PPP: Pentose phosphate pathway
OT: Oxythiamine
Pdhb: Pyruvate dehydrogenase subunit beta e1
RORE: ROR response element
RPIA: Ribose-5-phosphate isomerase
SOD2: Manganese superoxide dismutase 2
Timm23: Translocase of inner mitochondrial membrane 23
TKT: Transketolase
TOMM20: Translocase of outer mitochondrial membrane 20
WT: Wild type

## References

[1] Fontaine C (2003). The orphan nuclear receptor Rev-Erbalpha is a peroxisome proliferator-activated receptor (PPAR) gamma target gene and promotes PPARgamma-induced adipocyte differentiation. J. Biol. Chem..

[2] Preitner N (2002). The orphan nuclear receptor REV-ERBalpha controls circadian transcription within the positive limb of the mammalian circadian oscillator. Cell.

[3] Raspe E (2002). Identification of Rev-erbα as a physiological repressor of apoC-III gene transcription. J. Lipid Res..

[4] Mohwak JA, Green CB, Takahashi JS (2012). Central and peripheral circadian clocks in mammals. Annu. Rev. Neurosci..

[5] Zhang Y (2015). GENE REGULATION. Discrete functions of nuclear receptor Rev-erbalpha couple metabolism to the clock. Science.

[6] Duez H, Staels B (2008). Rev-erb alpha gives a time cue to metabolism. FEBS Lett..

[7] Solt LA (2012). Regulation of circadian behavior and metabolism by synthetic REV-ERB agonists. Nature.

[8] Amador A (2018). Distinct roles for REV-ERBα and REV-ERBβ in oxidative capacity and mitochondrial biogenesis in skeletal muscle. Plos One.

[9] Woldt E (2013). Rev-erb-α modulates skeletal muscle oxidative capacity by regulating mitochondrial biogenesis and autophagy. Nat. Med..

[10] Dyar KA (2018). Transcriptional programming of lipid and amino acid metabolism by the skeletal muscle circadian clock. PLOS Biology.

[11] Zhang Y (2016). HNF6 and Rev-erbα integrate hepatic lipid metabolism by overlapping and distinct transcriptional mechanisms. Genes Dev..

[12] Ikeda R (2019). REV-ERBα and REV-ERBβ function as key factors regulating mammalian circadian output. Sci. Rep..

[13] Sengupta S (2016). The circadian gene Rev-erbα improves cellular bioenergetics and provides preconditioning for protection against oxidative stress. Free Radical Biol. Med..

[14] Patra KC, Hay N (2014). The pentose phosphate pathway and cancer. Trends Biomed. Sci..

[15] Boros LG (1997). Oxythiamine and dehydroepiandrosterone inhibit the nonoxidative synthesis of ribose and tumor cell proliferation. Can. Res..

[16] Popovici T, Berwald-Netter Y, Vibert M, Kahn A, Skala H (1990). Localization of aldolase C mRNA in brain cells. FEBS Lett..

[17] Thompson RJ, Kyonch PAM, Willson VJC (1981). Cellular localization of adolase C subunits in the human brain. Brain Res..

[18] Han Z, Zhong L, Srivastava A, Stacpoole PW (2008). Pyruvate dehydrogenase complex deficiency caused by ubiquitination and proteasome-mediated degradation of the E1β subunit. J. Biol. Chem..

[19] Fang R, Nixon PF, Duggelby RG (1998). Identification of the catalytic glutamate in the E1 component of human pyruvate dehydrogenase. FEBS Lett..

[20] Yin L, Wang J, Klein PS, Lazar MA (2006). Nuclear Receptor Rev-erba is a critical lithium sensing component of the circaidian clock. Science.

[21] Lee CR, Park YH, Min H, Kim YR, Seok YJ (2019). Determination of protein phosphorylation by polyacrylamide gel electrophoresis. J. Microbiol..

[22] Wegener AD, Jones LR (1984). Phosphorylation-induced mobility shift in phospholamban in sodium dodecyl sulfate-polyacrylamide gels. J. Biol. Chem..

[23] Benito A (2017). Glucose-6-phosphate dehydrogenase and transketolase modulate breast cancer cell metabolic reprogramming and correlate with poor patient outcome. Oncotarget.

[24] Qiu Z (2015). MicroRNA-124 reduces the pentose phosphate pathway and proliferation by targeting PRPS1 and RPIA mRNAs in human colorectal cancer cells. Gastroenterology.

[25] Ciou SC (2015). Ribose-5-phosphate isomerase A regulates hepatocarcinogenesis via PP2A and ERK signaling. Int. J. Cancer.

[26] Boros LG (1997). Oxythiamine and dehydroepiandrosterone inhibit the nonoxidative synthesis of ribose and tumor cell proliferation. Can. Res..

[27] Rais B (1999). Oxythiamine and dehydroepiandrosterone induce a G1 phase cycle arrest in Ehrlich's tumor cells through inhibition of the pentose cycle. FEBS Lett..

[28] Wang J (2013). Inhibition of transketolase by oxythiamine altered dynamics of protein signals in pancreatic cancer cells. Exp. Hematol. Oncol..

[29] Aft RL, Zhang FW, Gius D (2002). Evaluation of 2-deoxy-d-glucose as a chemotherapeutic agent: mechanism of cell death. Br. J. Cancer.

[30] Wick AR, Drury DR, Nakada HI, Wolfe JB (1956). Localization of the primary metabolic block produced by 2-deoxyglucose. J. Biol. Chem..

[31] Yang G (2014). Oxidative stress and inflammation modulate Rev-erbα signaling in the neonatal lung and affect circadian rhythmicity. Antioxid. Redox Signal..

[32] Janzen EG, Jandristis LT, Shetty RV, Haire DL, Hilborn JW (1989). Synthesis and purification of 5,5-dimethyl-1-pyrroline-N-oxide for biological applications. Chem. Biol. Interact..

[33] Ranguelova K, Mason RP (2011). The fidelity of spin trapping with DMPO in biological systems. Magn. Reson. Chem..

[34] Frankel D, Mehindate K, Schipper HM (2000). Role of heme oxygenase-1 in the regulation of manganese superoxide dismutase gene expression in oxidatively-challenged astroglia. J. Cell. Physiol..

[35] Gregory EM, Goscin SA, Fridovich I (1974). Superoxide dismutase and oxygen toxicity in a eukaryote. J. Bacteriol..

[36] Vile GF, Basu-Modak S, Waltner C, Tyrrell RM (1994). Heme oxygenase 1 mediates an adaptive response to oxidative stress in human skin fibroblasts. Proc. Natl. Acad. Sci. USA.

[37] Dierichkx P (2019). SR9009 has REV-ERB–independent effects on cell proliferation and metabolism. Proc. Natl. Acad Sci. USA.

[38] Zhang T (2018). REV-ERBα regulates CYP7A1 through repression of liver receptor homolog-1. Drug Metab. Dispos..

[39] Zhang Y (2016). HNF6 and Rev-erbalpha integrate hepatic lipid metabolism by overlapping and distinct transcriptional mechanisms. Genes Dev..

[40] Toledo M (2018). Autophagy regulates the liver clock and glucose metabolism by degrading CRY1. Cell Metab..

[41] Yuan X, Dong D, Li Z, Wu B (2019). Rev-erbα activation down-regulates hepatic Pck1 enzyme to lower plasma glucose in mice. Pharmacol. Res..

[42] Delezie J (2012). The nuclear receptor REV-ERBa is required for the daily balance of carbohydrate and lipid metabolism. FASEB J..

[43] Gibbs JE (2012). The nuclear receptor REV-ERBα mediates circadian regulation of innate immunity through selective regulation of inflammatory cytokines. Proc. Natl. Acad. Sci. U.S.A.

[44] Amir M (2018). REV-ERBα regulates TH17 cell development and autoimmunity. Cell Rep..

[45] Chang C (2019). The nuclear receptor REV-ERBα modulates Th17 cell-mediated autoimmune disease. Proc. Natl. Acad. Sci. USA.

[46] Fuhr L (2018). The circadian clock regulates metabolic phenotype rewiring via HKDC1 and modulates tumor progression and drug response in colorectal cancer. EBioMedicine.

[47] Hunter AL (2020). Nuclear receptor REVERBalpha is a state-dependent regulator of liver energy metabolism. Proc. Natl. Acad. Sci. USA.

[48] Balsalobre A, Damiola F, Schibler U (1998). A serum shock induces circadian gene expression in mammalian tissue culture cells. Cell.

[49] Ling L (2018). Transcriptional regulation of the Warburg effect in cancer by SIX1. Cancer Cell.

[50] Weber G (1977). Enzymology of cancer cells. N. Engl. J. Med..

[51] Warburg O (1956). On the origin of cancer cells. Science.

[52] Sulli G (2018). Pharmacological activation of REV-ERBs is lethal in cancer and oncogene-induced senescence. Nature.

[53] Burgermeister E (2019). Aryl hydrocarbon receptor nuclear translocator-like (ARNTL/BMAL1) is associated with bevacizumab resistance in colorectal cancer via regulation of vascular endothelial growth factor A. EBioMedicine.

[54] Wagner PM, Monjes NM, Guido ME (2019). Chemotherapeutic Effect of SR9009, a REV-ERB Agonist, on the Human Glioblastoma T98G Cells. ASN Neuruo.

[55] Na H (2019). High expression of NR1D1 is associated with good prognosis in triple-negative breast cancer patients treated with chemotherapy. Breast Cancer Res..

[56] Ka N, Na T, Lee M (2017). NR1D1 enhances oxidative DNA damage by inhibiting PARP1 activity. Mol. Cell. Endocrinol..

[57] Tao L (2019). Rev-erbalpha inhibits proliferation by reducing glycolytic flux and pentose phosphate pathway in human gastric cancer cells. Oncogenesis.

[58] Chu G, Zhou Q, Hu Y, Shengjie S, Yang G (2019). Rev-erb inhibits proliferation and promotes apoptosis of preadipocytes through the agonist GSK4112. Int. J. Mol. Sci..

[59] Bustamante E, Pedersen PL (1977). High aerobic glycolysis of rat hepatoma cells in culture: role of mitochondrial hexokinase. Proc. Natl. Acad. Sci. U.S.A..

[60] Patra KC (2013). Hexokinase 2 is required for tumor initiation and maintenance and its systemic deletion is therapeutic in mouse models of cancer. Cancer Cell.

[61] Wang L (2014). Hexokinase 2-mediated warburg effect is required for PTEN- and p53-deficiency-driven prostate cancer growth. Cell Rep..

[62] Taro H (2010). Tyrosine phosphorylation inhibits PKM2 to promote the Warburg effect and tumor growth. Sci. Signal..

[63] Christofk HR (2008). The M2 splice isoform of pyruvate kinase is important for cancer metabolism and tumour growth. Nature.

[64] Wang J (2016). The platelet isoform of phosphofructokinase contributes to metabolic reprogramming and maintains cell proliferation in clear cell renal cell carcinoma. Oncotarget.

[65] Li L (2018). TAp73-induced phosphofructokinase-1 transcription promotes the Warburg effect and enhances cell proliferation. Nat. Commun..

[66] Yao H (2019). Fatty acid oxidation protects against hyperoxia-induced endothelial cell apoptosis and lung injury in neonatal mice. Am. J. Respir. Cell Mol. Biol..

[67] Gong J (2021). The pentose phosphate pathway mediates hyperoxia-induced lung vascular dysgenesis and alveolar simplification in neonates. JCI Insight.

