# Supplementary material for: Loss of the transcriptional repressor Rev-erbα upregulates metabolism and proliferation in cultured mouse embryonic fibroblasts
Source: Sci Rep. 2021 Jun 11;11:12356. doi: 10.1038/s41598-021-91516-5 (PMC8196003; doi:10.1038/s41598-021-91516-5)

Supplemental document

Loss of the transcriptional repressor Rev-erbα upregulates metabolism and proliferation in cultured mouse embryonic fibroblasts

Sean P. Gillis[1#], Hongwei Yao[1#], Salu Rizal[1], Hajime Maeda[1], Julia Chang[1], Phyllis A. Dennery[1,2 *]

[1]Department of Molecular Biology, Cellular Biology and Biochemistry, Brown University, Providence, RI; [2]Department of Pediatrics, Warren Alpert Medical School of Brown University, Providence, RI

***To whom correspondence should be addressed**:
Phyllis A. Dennery, M.D.,
Department of Pediatrics
and Molecular Biology, Cellular Biology, and Biochemistry,
Brown University,
Department of Pediatrics
593 Eddy St Suite 125
Providence, RI 02903
Tel: 401-444-5648
Fax: 401-444-6378
Email: phyllis_dennery@brown.edu;

[#]These authors contributed equally to this work.

Supplemental Figure 1

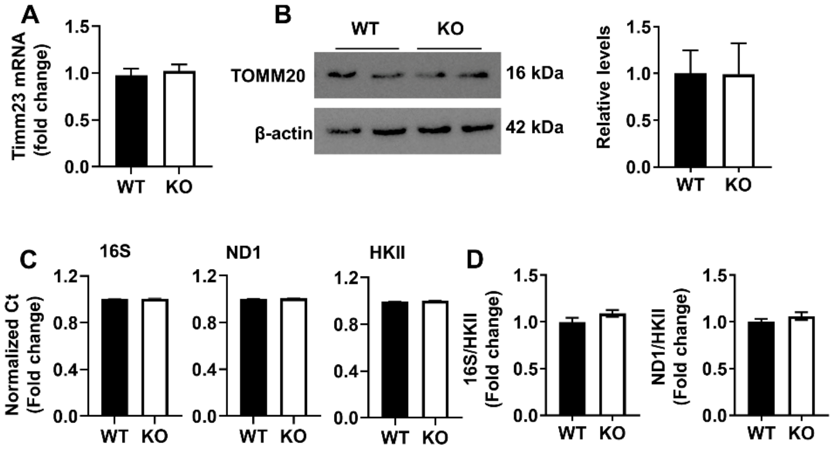

Supplemental Fig 1. **Loss of Rev-erbα does not change mitochondrial numbers, mitochondrial mass, or mtDNA content**. (A) Expression of Timm23 gene in WT and Rev-erbα KO MEFs. (B) Western blot for levels of the outer mitochondrial protein TOMM20 in WT and Rev-erbα KO MEFs. n = 9 independent experiments. (C, D) Mitochondrial DNA including 16S and ND1 was measured by PCR and normalized into nuclear DNA hexokinase II (HKII) in WT and Rev-erbα KO MEFs. n=3 independent experiments.

Supplemental Figure 2

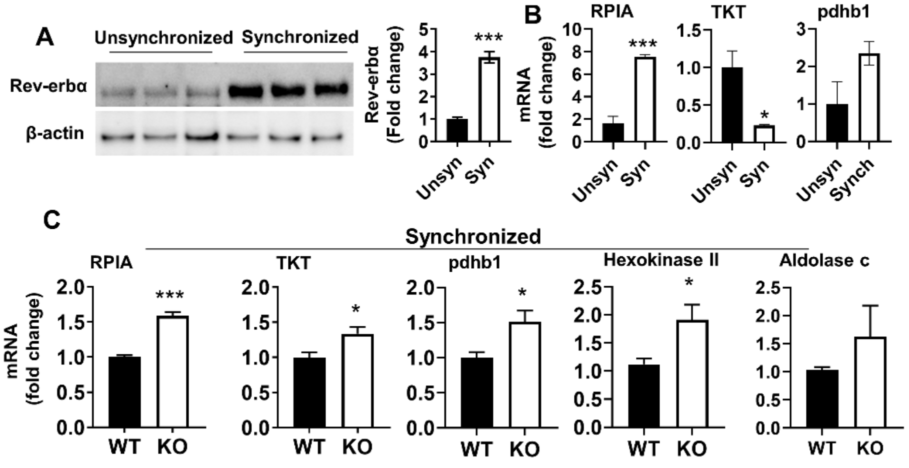

Supplemental Fig 2. **Synchronization increases Rev-erbα protein levels and Rev-erbα deletion increases expression of genes encoding enzymes in glycolysis and the PPP in synchronized MEFs**. (A, B) WT MEFs were cultured in medium without FBS for 24 hours. Rev-erbα protein was measured by Western blot (A), while RPIA, TKT and pdhb1 genes were measured by qRT-PCR (B). n=3-6 independent experiments. (C) Expression of RPIA, TKT, pdhb1, hexokinase II, and aldolase c was measured by qRT-PCR in WT and Rev-erbα KO MEFs cultured in medium without FBS. n = 3 independent experiments. Error bars represented as mean ± SEM. $^{*}p<0.05$, $^{***}p<0.001$ vs WT.

Supplemental Figure 3

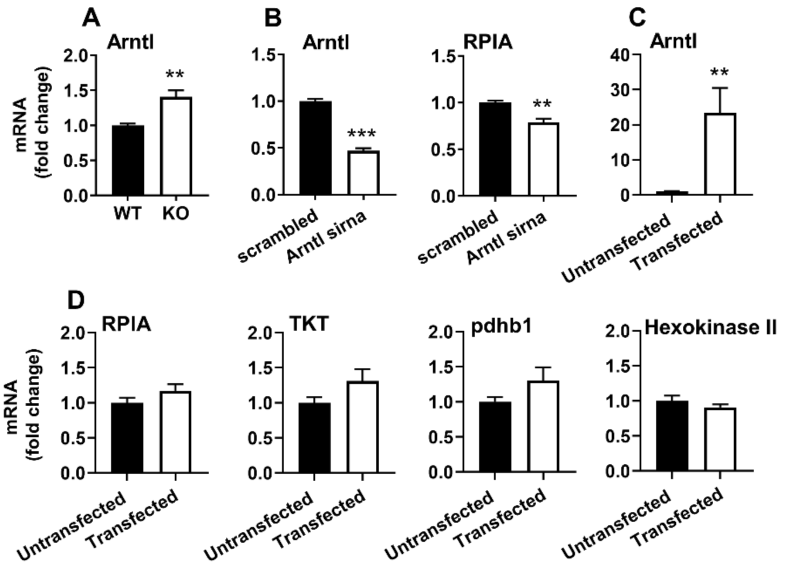

Supplemental Fig 3. Rev-erbα does not regulate target genes through BMAL1. (A) Expression of arntl, the gene encoding BMAL1, in WT and Rev-erbα KO MEFs. (B) Expression of RPIA gene following siRNA mediated knockdown of arntl in Rev-erbα KO background. (C) Expression of arntl gene following plasmid mediated arntl overexpression in WT MEFs. (D) Expression of RPIA, TKT, pdhb1, and hexokinase II genes in MEFs transfected with arntl plasmids in WT MEFs. n = 3 independent experiments. Error bars represented as mean ± SEM. $^{**}p < 0.01$, $^{***}p < 0.001$ vs WT (A), scrambled (B) or untransfected (C, D).

Supplemental Figure 4

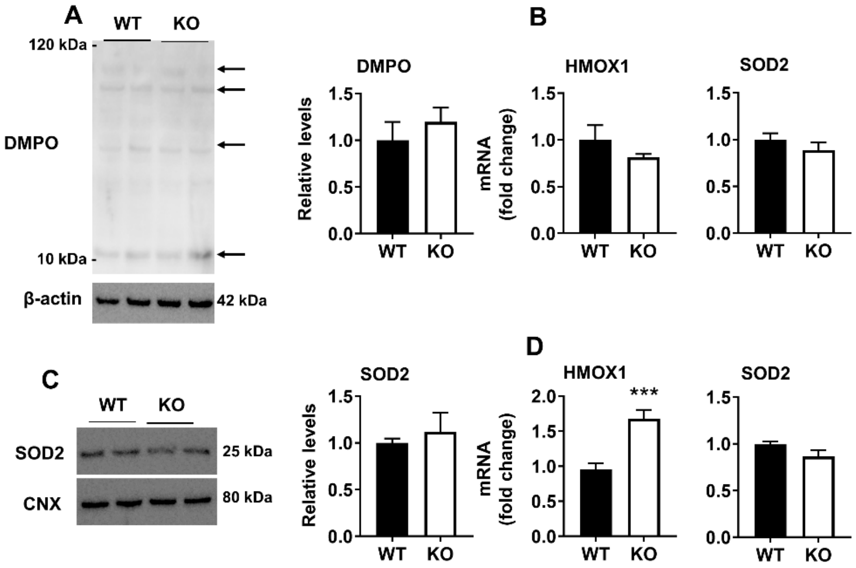

Supplemental Fig 4. Rev-erbα does not influence basal levels of oxidative stress. (A) Densitometry analysis of total 5,5-dimethyl-1-pyrroline N-oxide protein adducts normalized to β-actin levels in WT and Rev-erbα KO MEFs measured by Western blot. (B) Expression of HMOX1 and SOD2 genes in WT and Rev-erbα KO MEFs. (C) SOD2 protein levels measured by Western blot in WT and Rev-erbα KO MEFs. SOD2 levels were normalized to levels of a calnexin (CNX) loading control. (D) Expression of HMOX1 and SOD2 genes in WT and Rev-erbα KO MEFs following 24 hours of 500 µM H_2O_2. For qPCR, n = 3 independent experiments; for immunoblotting n = 8-9 independent experiments. Error bars represented as mean ± SEM. $^{***}p < 0.001$ vs WT.

Full images of Western blot in Figure 1B and 3A

Full images of Western blot

Figure 1B

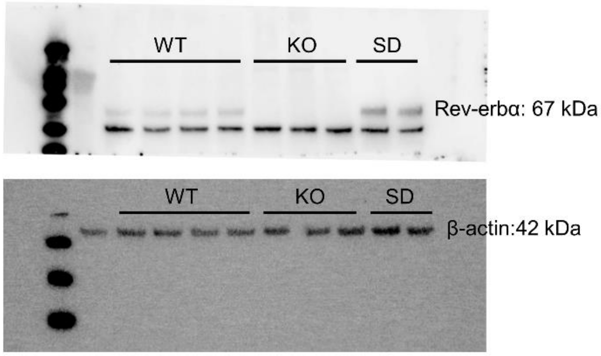

Figure 3A

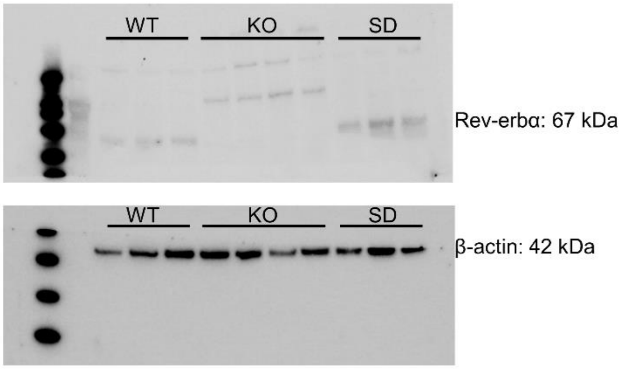

Full images of Western blot in Figure 4G

Full images of Western blot

Figure 4G

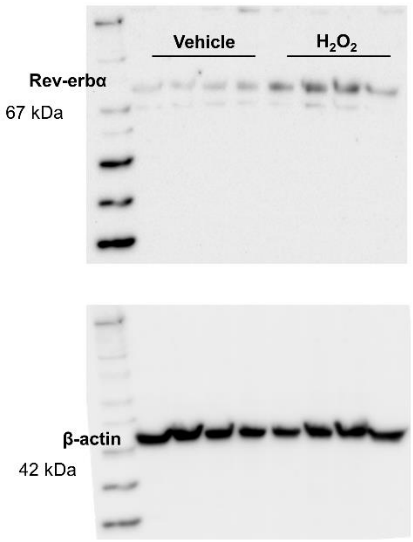

Full images of Western blot in Supplemental Figure 1B and 2A

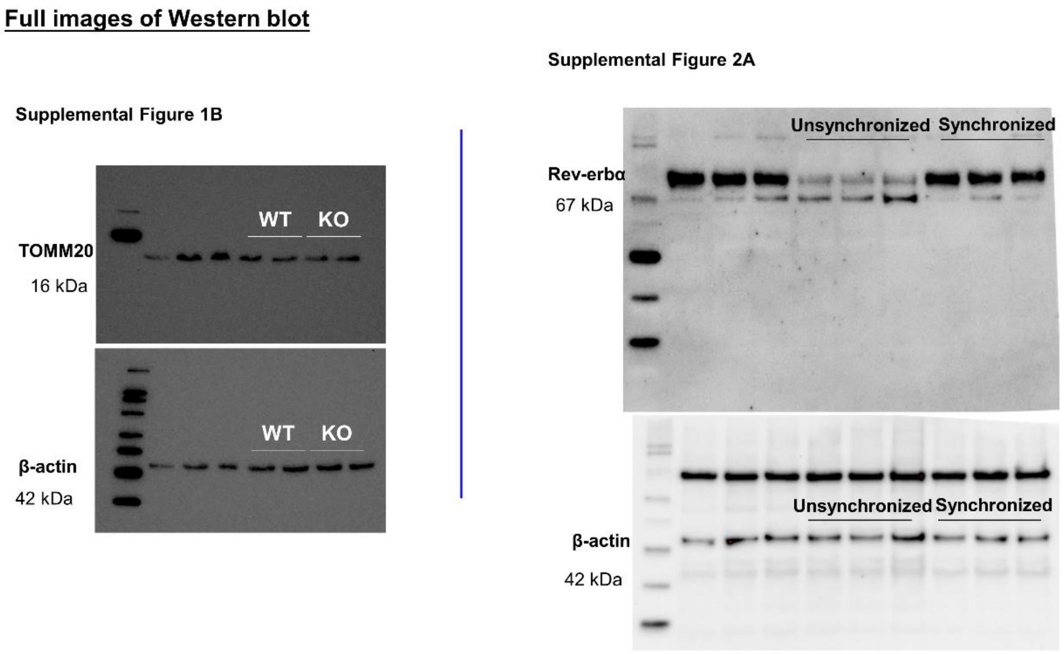

Full images of Western blot in Supplemental Figure 4A and 4C

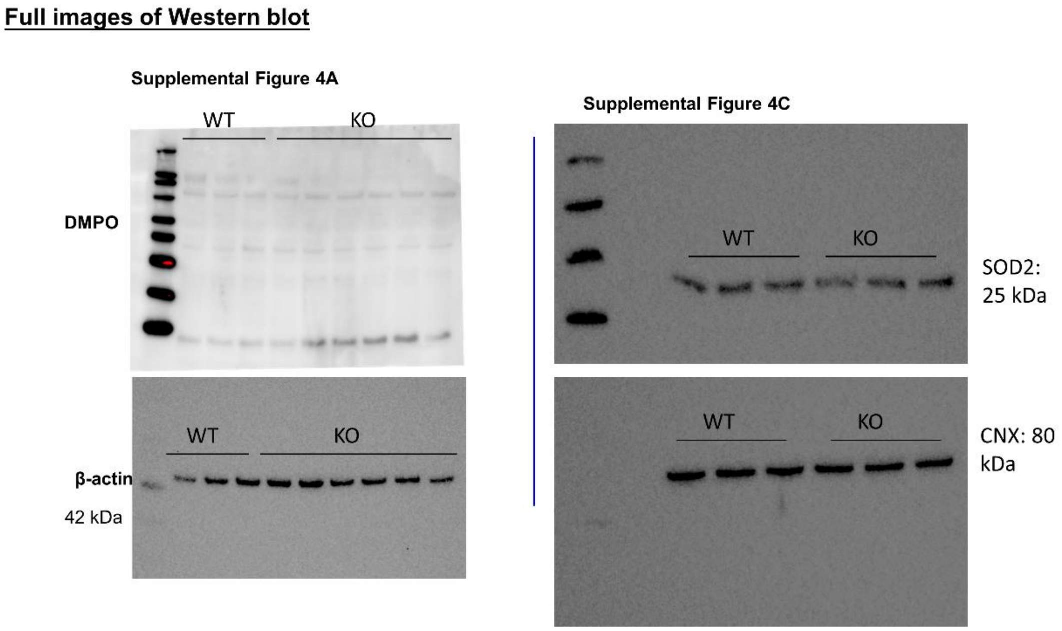

Supplement: Supplementary file 1 — Supplementary Information. [file 41598_2021_91516_MOESM1_ESM.pdf]
